# New Dermoscopic Keys for Circumscribed Acral Hypokeratosis: Report of Four Cases

**DOI:** 10.5826/dpc.1102a10

**Published:** 2021-03-08

**Authors:** Paula Majluf-Cáceres, Cristián Vera-Kellet, Sergio González-Bombardiere

**Affiliations:** 1 Department of Dermatology, Pontificia Universidad Católica de Chile; 2 Department of Dermatopathology, Pontificia Universidad Católica de Chile

**Keywords:** acral hypokeratosis, image enhancement, dermatopathology, dermoscopy

## Introduction

Descriptions of the dermoscopic features of acral hypokeratosis (AH) are few. Clinically it can resemble other entities, such as Bowen disease or porokeratosis of Mibelli. Although AH is considered a benign pathology, in 2010 a case with actinic keratosis in the hypokeratosic epidermis and underlying elastosis was reported [1], hence the importance of knowing the dermoscopic findings for an early diagnosis and to rule out other differential or coexisting diagnoses.

## Case Presentation

**Figure 1:**
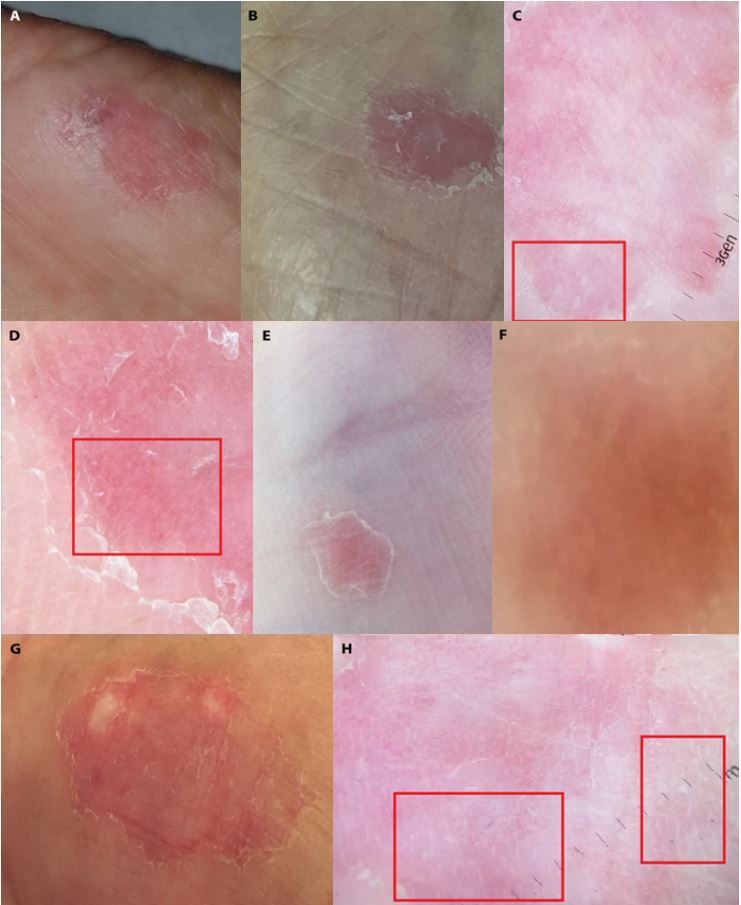
Clinical and dermoscopic features. (A and B) Cases 1 and 2: Atrophic erythematous plaque with an irregular hyperkeratotic border. (C) Dermoscopy shows pink areas on a red milky blush with scattered red dots, step-like scales at the periphery and elongated whitish structures in a raindrop pattern. (D) Dermoscopy shows red milky blush, pink islets with dotted vessels, elongated whitish structures in raindrop pattern and staircase sign. (E) Case 3: Depressed erythematous plaque, surrounded by an hyperkeratotic border. (F) Dermoscopy showed a red dot pattern over a homogeneous red-yellow area.

Our case series was comprised of 4 patients with AH confirmed by biopsy in the hypothenar eminence. Figure 1A shows AH in a 61-year-old and Figure 1B a 78-year-old woman with a 10-year history of AH. Dermoscopy revealed pink areas on a red milky blush with scattered red dots, step-like scales at the periphery, and elongated whitish structures in a fibrillar raindrop pattern (Figure 1, C and D).

**Figure 2:**
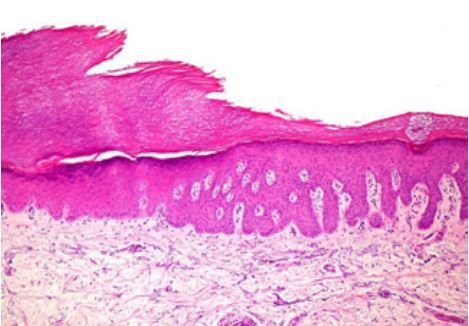
Histology displays an area of hypokeratosis demarcated by a sharp and frayed cut-off from uninvolved acral skin with discrete hypogranulosis, dilated blood vessels in the papillary dermis and slightly thickened collagen fibers in the reticular dermis (H&E, ×10).

The third case corresponded to asymptomatic AH (Figure 1E) that had developed 2 weeks after a cutting wound in a 30-year-old woman. Dermoscopy showed a red dot pattern over a homogeneous red-yellow area (Figure 1F). The fourth case was a 54-year-old woman affected by AH for 8 years (Figure 1G). Dermoscopy revealed a fine white pseudonetwork, pink stiff areas on a red milky blush with red dots, step-like scales at the periphery, and elongated whitish structures in a fibrillar raindrop pattern. (Figure 1H). Microscopy revealed an area of hypokeratosis demarcated by a sharp and frayed cut-off from uninvolved acral skin with discrete hypogranulosis, dilated blood vessels in the papillary dermis, and slightly thickened collagen fibers in the reticular dermis (Figure 2).

## Conclusions

Previous case series reported star-like desquamation at the periphery, and a well-demarcated erythema with reddish dots. These structures correlate with histopathological studies showing a sharply demarcated area of hypokeratosis, dilated capillaries in the papillary dermis and vessels in the upper reticular dermis [2]. A recent case report of congenital plantar AH showed a white thin scale and a reticulated surface with no visible acrosyringia opposing the typical dermoscopic acral pattern [3].

Our study revealed different dermoscopic findings than previously published: A fine white pseudonetwork and elongated whitish structures in a “raindrop pattern” found in those patients with longstanding AH and could be correlated with the increasing collagen proliferation and thickening. Thus in our case of 2-weeks’ onset AH, only a yellowish-red blush and red dots with peripheral step-like scales were distinguishing.
